# Dysbiosis index and fecal concentrations of sterols, long-chain fatty acids and unconjugated bile acids in dogs with inflammatory protein-losing enteropathy

**DOI:** 10.3389/fmicb.2024.1433175

**Published:** 2024-10-11

**Authors:** Federica Cagnasso, Jan S. Suchodolski, Antonio Borrelli, Franca Borella, Enrico Bottero, Elena Benvenuti, Riccardo Ferriani, M. Katherine Tolbert, Chih-Chun Chen, Paula R. Giaretta, Paola Gianella

**Affiliations:** ^1^Department of Veterinary Sciences, University of Turin, Grugliasco, Italy; ^2^Gastrointestinal Laboratory, Department of Small Animal Clinical Sciences, Texas A&M University, College Station, TX, United States; ^3^Associazione Professionale Endovet, Rome, Italy

**Keywords:** canine, fecal long-chain fatty acids, fecal sterols, fecal bile acids, dysbiosis

## Abstract

**Introduction:**

Canine protein-losing enteropathy (PLE) is a syndrome characterized by gastrointestinal loss of proteins. While fecal microbiome and metabolome perturbations have been reported in dogs with chronic enteropathy, they have not been widely studied in dogs with PLE. Therefore, the study aims were to investigate gut microbiome and targeted fecal metabolites in dogs with inflammatory PLE (iPLE) and evaluate whether treatment affects these changes at short-term follow-up.

**Methods:**

Thirty-eight dogs with PLE and histopathological evidence of gastrointestinal inflammation and 47 healthy dogs were enrolled. Fecal samples were collected before endoscopy (T0) and after one month of therapy (T1). Microbiome and metabolome alterations were investigated using qPCR assays (dysbiosis index, DI) and gas chromatography/mass spectrometry (long-chain fatty acids, sterols, unconjugated bile acids), respectively.

**Results:**

Median (min-max) DI of iPLE dogs was 0.4 (−5.9 to 7.7) and was significantly higher (*p* < 0.0001) than median DI in healthy dogs [−2.0 (−6.0 to 5.3)]. No significant associations were found between DI and selected clinicopathological variables. DI did not significantly differ between T0 and T1. In iPLE dogs, at T0, myristic, palmitic, linoleic, oleic, cis-vaccenic, stearic, arachidonic, gondoic, docosanoic, erucic, and nervonic acids were significantly higher (*p* < 0.0001) than healthy dogs. In iPLE dogs, oleic acid (*p* = 0.044), stearic acid (*p* = 0.013), erucic acid (*p* = 0.018) and nervonic acid (*p* = 0.002) were significantly decreased at T1. At T0, cholesterol and lathosterol (*p* < 0.0001) were significantly higher in iPLE dogs compared to healthy dogs, while total measured phytosterols were significantly lower (*p* = 0.001). No significant differences in total sterols, total phytosterols and total zoosterols content were found at T1, compared to T0. At T0, total primary bile acids and total secondary bile acids did not significantly differ between healthy control dogs and iPLE dogs. No significant differences in fecal bile acid content were found at T1.

**Discussion:**

Dysbiosis and lipid metabolism perturbations were observed in dogs with iPLE. Different therapeutic protocols lead to an improvement of some but not all metabolome perturbations at short-term follow-up.

## 1 Introduction

Protein-losing enteropathy (PLE) is a complex and challenging syndrome, characterized by chronic gastrointestinal signs and abnormal loss of proteins through the gastrointestinal tract (Jergens and Heilmann, 2022; Allenspach and Iennarella-Servantez, 2021; Green and Kathrani, 2022; Craven and Washabau, 2019). Numerous gastrointestinal diseases including inflammatory enteropathy, lymphangiectasia, and neoplasia, if severe enough, can result in PLE (Craven and Washabau, 2019). The diagnosis of the intestinal disorder causing PLE is time-consuming, the management is challenging, and the prognosis is guarded, with death occurring in > 50% of dogs with inflammatory PLE (Allenspach and Iennarella-Servantez, 2021; Green and Kathrani, 2022; Craven and Washabau, 2019). Therefore, a timely and early diagnosis and therapeutic intervention are desirable.

At present, the pathogenesis of PLE is not fully understood and appears to be multifactorial (Jergens and Heilmann, 2022; Craven and Washabau, 2019). Several markers have been described as negative prognostic factors of PLE, including hypocobalaminemia, increased CRP, and elevated CCECAI, although they are inconsistently observed during the course of the disease and are not pathognomonic (Allenspach and Iennarella-Servantez, 2021). It is suspected that in the context of a multifactorial pathogenesis and a complex and intricate disease, the population of dogs affected by PLE may vary in terms of clinical and pathological aspects, also in relation to the severity and extent of mucosal damage.

The gut microbiome plays an important role in preserving the intestinal mucosal barrier function, educating the immune system, driving inflammation, and affecting most physiologic functions through the production of metabolites (Tizard and Jones, 2018; Pilla and Suchodolski, 2020). Indeed, a dysfunctional microbiome, as observed in many acute and chronic gastrointestinal diseases, is associated with dysbiosis (Jergens and Heilmann, 2022; Allenspach and Iennarella-Servantez, 2021; Green and Kathrani, 2022). Recently, a quantitative PCR-based assay, namely the dysbiosis index (DI), was developed to assess shifts in the microbiome in fecal samples of dogs (AlShawaqfeh et al., 2017). It quantifies the fecal abundance of seven core bacteria and combines them into a single numeric value that accurately predicts global shifts in the canine microbiome as assessed by metagenomic sequencing (Sung et al., 2023a). The selected taxa of the DI that are frequently altered in a subset of dogs with chronic enteropathies have important metabolic functions for the host (AlShawaqfeh et al., 2017).

The intestinal metabolome is the biochemical environment representing a symbiosis between the host and the microbiota, broadly reflecting the health of the gastrointestinal tract (Pilla and Suchodolski, 2020; Bauset et al., 2021). The host provides a nutrient-rich environment, and the microbiota performs functions and produces metabolites. Chronic inflammatory enteropathy alters the fecal and serum metabolome, such as bile acids, amino acids, short- and long-chain fatty acids, vitamins, and their derivatives (Minamoto et al., 2015). Sterols and long-chain fatty acids are two important lipidic macronutrients that play different essential functions for the organism, such as energy production and storage, cellular membrane structure composition, and regulation of different biological processes, including the inflammation pathway (Piotrowska et al., 2021; Weng et al., 2019; Ma et al., 2019). Bile acids (BAs) play a key role in lipid absorption and metabolism and intestinal inflammatory processes. Primary conjugated bile acids are converted in the large intestine into secondary unconjugated bile acids, which exhibit beneficial properties for the intestinal functions and interact with the gut immune system (Pilla and Suchodolski, 2020; Ward et al., 2017; Kang et al., 2019).

Altered fecal concentrations of long-chain fatty acids, sterols, and bile acids have been observed in humans, cats, and dogs with gastrointestinal disease (Piotrowska et al., 2021; Ma et al., 2019; Blake et al., 2019; Marsilio et al., 2021; Sung et al., 2023b; Galler et al., 2022a; Galler et al., 2022b; Pilla et al., 2021). In particular, a subset of dogs with chronic inflammatory enteropathy have decreased fecal secondary bile acid concentrations compared to healthy dogs (Blake et al., 2019; Galler et al., 2022b; Honneffer, 2017; Xu et al., 2016; Ziese and Suchodolski, 2021), while a subset of Yorkshire terriers with chronic inflammatory enteropathy have increased fecal long-chain fatty acids, along with decreased plant sterol sitostanol (Galler et al., 2022b).

To the authors’ knowledge, scarce information of microbiome and metabolome perturbations is available in dogs with inflammatory PLE (iPLE) both before and after treatment. The study of fecal microbiome and metabolome in dogs with iPLE might provide new insights into the magnitude and significance of intestinal dysmetabolism and damage, and potentially guide new therapeutic approaches. Therefore, the aim of this study was to investigate the DI and the fecal concentrations of sterols, long-chain fatty acids and unconjugated bile acids in a population of dogs with PLE caused by inflammatory enteropathy, both at diagnosis and short-term follow-up.

## 2 Materials and methods

### 2.1 Study design and ethics approval

The experimental protocol was reviewed and approved by the Ethics and Animal Welfare committee of the University of Turin (protocol number 42, 08/01/2021). This was a prospective investigation that involved client-owned dogs, and all owners provided informed consent. All the dogs were referred for a specialist consult to the Unit of Gastroenterology at the Veterinary Teaching Hospital of the University of Turin or to Endovet referral clinics in north-middle Italy between January 2021 and March 2022.

### 2.2 Cases and control dogs

Thirty-eight privately-owned dogs with a diagnosis of iPLE were enrolled. Inclusion criteria for inflammatory PLE were chronic gastrointestinal signs lasting for more than 3 weeks, hypoalbuminemia of gastrointestinal origin (≤ 2.8 g/dL) and histopathological evidence of benign gastrointestinal inflammation with or without lymphangiectasia on multiple biopsies collected by endoscopy. The histopathologic evaluation was performed according to the standards of the World Small Animal Veterinary Association Gastrointestinal Standardization Group (Day et al., 2008). Fecal flotation and giardia antigen-test, complete blood count, biochemistry, pre- and post-prandial bile acids, urinalysis, urinary protein to creatinine ratio, serum basal cortisol or ACTH stimulation test (if basal cortisol < 2 μg/dl), trypsin-like-immunoreactivity, pancreas specific lipase levels, serum folate and cobalamin concentrations, and abdominal ultrasound examination were required to rule out infectious, parasitic, liver and pancreatic diseases, along with intestinal diseases of other etiology and extraintestinal diseases. Hypoalbuminemic dogs also were required to have no clinically relevant proteinuria (negative urine dipstick test result or urine protein to creatinine ratio < 0.5) and no evidence of clinically relevant hepatic disease (normal pre- and post-prandial bile acid concentrations or normal synthetic liver function and enzyme activity). Exclusion criteria were complete and sustained response to hydrolyzed or limited ingredient diets administered before referral, incomplete diagnostic investigations, and a histopathologic diagnosis of neoplasia. Dogs with iPLE that received antibiotics prior to referral were excluded. Information concerning age, sex, breed, weight, chronic canine enteropathy clinical activity index (CCECAI) score, type of diets and therapeutic treatments prior to referral were registered at admission (Allenspach et al., 2007). The CCECAI was calculated at the time of the consultation, or retrospectively, using the serum albumin concentration, presence or absence of peripheral edema and peritoneal effusion on ultrasound examination and the owner’s scores on appetite, activity level, vomiting, fecal consistency and frequency, weight loss and pruritus. All iPLE dogs underwent gastroduodenoscopy. Colonoscopy with ileal intubation was performed when possible, based on the dog’s overall risk for prolonged anesthesia due to complications associated with severe hypoalbuminemia. At least 8 endoscopic biopsies from the stomach, duodenum, and when available, ileum and colon, were collected and immediately placed in a tube filled with 10% neutral buffered formalin and submitted for histologic examination. The type and severity of structural and inflammatory lesions in the duodenum, ileum, and colon were recorded based on a 4-point grading scheme (0 = normal, 1 = mild lesions, 2 = moderate lesions, 3 = severe lesions) (Day et al., 2008). Presence or absence of lymphangiectasia was determined based on histopathological evaluation of the diameter of lacteals, with lacteals representing more than 25% of the width of the villous lamina propria were considered dilated and subclassified as mild (25–50% of villous width), moderate (51–75% of villous width), or severe (> 75% of villous width), according to the World Small Animal Veterinary Association guidelines (Day et al., 2008). After the endoscopic procedure and histologic diagnosis (T0), diet and therapy were adjusted on a case basis. All iPLE dogs were prescribed ultra-low fat (< 15g fat/Mcal ME) or hydrolyzed diets in addition to oral prednisolone (0.5–1 mg/kg, q 12–24 h). In some dogs, oral prednisolone was administered with oral chlorambucil (2–4 mg/m2, q24h). Weekly parenteral cobalamin supplementation was given to dogs with hypocobalaminemia or suboptimal serum cobalamin concentrations (serum cobalamin levels within normal range but at the lower limit of the reference range, i.e., < 400 ng/L), as previously described (Berghoff et al., 2013; Kather et al., 2020). Daily oral folate supplementation was given to dogs with hypofolatemia (200 mcg for dogs < 20 kg, and 400 mcg for dogs ≥ 20 kg, PO once daily for 4 weeks). Clopidogrel (2 mg/kg, PO once daily) was administered on case-by-case basis. Diet and therapeutic protocols were not changed for the following month. After one month of therapy (T1), all iPLE dogs were re-evaluated by the same clinician. The CCECAI scores were recorded, serum total protein, albumin, cholesterol, and C-reactive protein concentrations were measured. Additional diagnostic investigations were done on a case basis.

Fifty healthy owned-dogs, regularly vaccinated and receiving appropriate ecto-and endo-parasite preventive treatment, belonging to staff at the Veterinary Teaching Hospital of Turin University or that were presented at the same Veterinary Teaching Hospital and referral clinics of the study group for their annual check-up and vaccination, were enrolled as a control group. These dogs were considered healthy based on unremarkable history and physical examination, negative fecal flotation, and absence of any gastrointestinal sign within one year prior to enrollment. In addition, there was no history of antibiotic administration within 3 months prior to enrollment nor ongoing drug administration. Finally, most of the healthy control dogs were fed commercial nutritionally complete and balanced canine diets of different brands.

### 2.3 Sample collection and storage

Naturally passed feces of iPLE dogs were collected the day before the endoscopic procedure (T0) and after one month of therapy (T1). All owners were instructed to collect and immediately freeze (−20°C) fecal samples. Feces of healthy control dogs were collected in the same study period of iPLE dogs and immediately frozen at −20°C. Fecal samples, of both iPLE and healthy control dogs, collected at home were frozen at −80°C the day after collection and refrigeration at −20°C. All fecal samples were shipped with priority on dry ice to the Gastrointestinal Laboratory, Department of Small Animal Clinical Sciences, Texas A&M University, USA, for the analyses. The samples’ condition was recorded at the destination.

### 2.4 Fecal dysbiosis index

DNA extraction from 100 mg of each fecal sample was performed using the MoBio Power soil DNA isolation kit (PowerSoil, Mo Bio Laboratories, Carlsbad, CA, USA) according to the manufacturer’s instruction (AlShawaqfeh et al., 2017). The qPCR panel consisted of eight bacterial groups: total bacteria, *Faecalibacterium* spp., *Turicibacter* spp., *Escherichia coli*, *Streptococcus* spp., *Blautia* spp., *Fusobacterium* spp., and *Clostridium (Peptacetobacter) hiranonis*. The qPCR assays were performed according to a previously published protocol (AlShawaqfeh et al., 2017). The data obtained were expressed as the log DNA abundance (fg) for each bacterial group/10 ng of total isolated DNA. The abundance of the evaluated bacterial groups was used to calculate the DI according to a mathematical algorithm previously validated (AlShawaqfeh et al., 2017). Dysbiosis was classified as significant (DI > 2), mild to moderate (DI 0–2), minor changes (DI < 2 with individual bacterial groups outside the reference interval), and normal (DI < 2 with no shifts in the overall diversity of the intestinal microbiota).

### 2.5 Fecal metabolome analysis

A gas chromatography-mass spectrometry (GC-MS) quantitative assay was performed to assess concentrations of the following targeted fecal metabolites: long-chain fatty acids (i.e., palmitic acid, linoleic acid, α-linolenic acid, oleic acid, cis-vaccenic acid, stearic acid, arachidonic acid, gondoic acid, erucic acid, docosanoic acid, and nervonic acid), zoosterols (i.e., cholesterol, coprostanol, cholestanol, and lathosterol), phytosterols (i.e., β-sitosterol, brassicasterol, campesterol, fusosterol, sitostanol, and stigmasterol), and unconjugated bile acids (i.e., cholic acid, chenodeoxycholic acid, lithocholic acid, deoxycholic acid, and ursodeoxycholic acid). A previously described protocol was used (Galler et al., 2022b; Honneffer, 2017; Batta et al., 2002). Briefly, a lyophilized fecal sample weighing 10–14 mg was aliquoted into a glass centrifuge tube. Deuterated internal standards including d7-sitostanol, d6-cholesterol, d4-stearic acid, d4-cholestane, d4-cholic acid, and d4-lithocholic acid were added to each sample. After the addition of concentrated HCl, the samples were incubated at 65°C for 4 h. The samples were then dried under nitrogen gas, followed by a silylation reaction by adding Sylon HTP (Sigma-Aldrich, St. Louis, MO, USA) and 30 minutes incubation at 65°C. After incubation, samples were dried with nitrogen gas again, then hexane was added. After the mixtures were centrifuged, the supernatants were injected individually into Agilent 8890 GC coupled with a 5977B GC/MSD (Agilent Technologies, Santa Clara, California, USA). The mass spectrometer was operated in selected ion monitoring mode for quantitative analysis, and Agilent ChemStation (Agilent Technologies, Santa Clara, California, USA) was used for peak integration and concentration calculations (Galler et al., 2022b; Honneffer, 2017; Batta et al., 2002).

These data were exported and the recorded weight of lyophilized feces for each sample was used to calculate concentrations in micrograms or nanograms per milligram of lyophilized feces. Data for the assessment of bile acids were reported as total amounts in nanograms per milligram of lyophilized fecal content and as percent of total bile acids measured. Total primary bile acids (TPBA) comprise the sum of cholic acid and chenodeoxycholic acid; total secondary bile acids (TSBA) comprise the sum of lithocholic acid, deoxycholic acid, and ursodeoxycholic acid. Total bile acids represent the sum of all measured bile acids. The percentage of TPBA% and TSBA%, which referred to the sum of CA and CDCA divided by the total measured BAs and the sum of LCA, DCA, and UDCA divided by the total measured BAs, were also calculated. Total measured phytosterols comprise the sum of β-sitosterol, brassicasterol, fusosterol, campesterol, sitostanol, and stigmasterol. Total measured zoosterols comprise the sum of cholesterol, coprostanol, cholestenol, and lathosterol. Total measured sterols comprise the sum of phyto- and zoosterols. Total measured fatty acid concentrations (FAs) comprise the sum of all measured long-chain fatty acids. Data for the assessment of sterols and FAs were reported as micrograms per milligram lyophilized feces.

The quality control and positive control used in this study were described earlier (Honneffer, 2017). In brief, quality control was implemented using a series of five samples: a preparation blank (PB), a zero blank (ZB), a sterol continuing calibration verification (S-CCV), a fatty acid continuing calibration verification (F-CCV), and a laboratory control sample (LCS). The PB contained only butanol and hydrochloric acid and ZB, similar to the PB but with the addition of internal standards were served to monitor contamination throughout preparation and analysis. The S-CCV and F-CCV were mixtures of stock solutions with known concentrations of sterols and fatty acids, respectively, to confirm accuracy. The LCS consisted of pooled and lyophilized fecal samples, ground and stored frozen to ensure consistency in analytical procedures.

### 2.6 Statistical analysis

All data were analyzed with the software GraphPad Prism 9 (Dotmatics). Data were tested for normal distribution using Shapiro-Wilk test. Comparisons of sex and breed between iPLE and healthy control dogs were evaluated using Fisher’s exact tests. Comparisons of quantitative clinicopathological variables, qPCR bacterial abundance, and fecal metabolite concentration between iPLE and healthy control dogs were analyzed using the Student’s *t*-test for normally distributed data and the Mann-Whitney *U*-test for non-normally distributed data. The same statistical tests were used whenever quantitative variables needed to be compared between two groups, depending on their distribution. Comparisons of quantitative clinicopathological variables, qPCR bacterial abundance, and fecal metabolite concentrations in iPLE dogs between T0 and T1 were performed using a paired *t*-test or Wilcoxon matched-pairs signed rank test, depending on their distribution. Furthermore, fold changes between T0 and T1 for each fecal metabolite studied, DI and each single bacterial taxa, have been calculated. Fold change was determined as the ratio of T1 to T0 fecal content, with a fold change greater than 1 indicating an increase in metabolite abundance and a fold change less than 1 indicating a decrease in metabolite abundance. Fold changes greater than 2 or less than 0.5 were considered biologically relevant. The One-Way ANOVA or Kruskal-Wallis test was used, based on variable distribution, for comparisons of quantitative variables in cases of groups ≥ 3. All fecal metabolites studied were compared between the various diet groups consumed prior to inclusion at T0 and they were also compared between the diet groups prescribed between T0 and T1. Spearman or Pearson rank tests were used to test correlations between the abundance of bacterial taxa and fecal concentrations of all the fecal metabolites studied. Spearman’s test was applied when the variables were not normally distributed (r_p_ = Pearson’s correlation coefficient), while Pearson’s test was used for variables that followed a normal distribution (r_s_ = Spearman’s correlation coefficient). Correlations between clinicopathological variables and bacterial abundance or targeted metabolites were also tested with the same approach.

Significance was set at *p* < 0.05. The *p*-values were adjusted with Bonferroni correction to account for multiple comparisons.

Principal coordinate analysis and hierarchical clustering heatmaps were generated using Metaboanalyst 5.0 based on the log-transformed with Pareto scaling data. To generate the PCA plot, the MetaboAnalyst software used the PERMANOVA test (Permutational Multivariate Analysis of Variance).

## 3 Results

### 3.1 Animals

Five out of fifty healthy control dogs had an increased DI (> 2), and at re-check with the owner it was discovered that 3 of them showed sporadic gastrointestinal symptoms, and all 3 dogs were therefore excluded from analysis.

The final study population consisted of 47 healthy control dogs and 38 dogs with iPLE. Eight dogs of the iPLE group were mixed breed (21.1%) and 30 dogs were purebred (78.9%), represented as follows: German Shepherd (6 dogs), Border Collie (3 dogs), Rottweiler, Belgian Shepherd, Chihuahua, Golden Retriever (2 dogs each breed), Cavalier King Charles, Dachshund, American Staffordshire Terrier, Australian Shepherd, English Setter, Maltese, Pitbull, French Bulldog, Podenco, Labrador Retriever, Yorkshire Terrier, Spanish Greyhound, Cesky Terrier (1 dog each breed). The control group included 19 mixed breed dogs (40%) and 28 pure breed dogs (represented by seventeen different breeds). Age, sex, and body weight did not significantly differ between healthy control and iPLE dogs.

At T0, the iPLE dogs showed the following median (range) values: CCECAI score 8 (3–17), total protein 4.1 g/dL (2–7.4), albumin 1.8 g/dL (0.9–2.7), cholesterol 116 mg/dL (63–327), and C-reactive protein 64.6 mg/L (0–48.4). Cobalamin and folate were measured in 24 out of 38 dogs with iPLE. Twelve iPLE dogs (50%) out of 24 had serum cobalamin concentrations lower than the reference interval, 6 (25%) had suboptimal serum cobalamin levels (normal but < 400 ng/L), and the remaining 6 dogs (25%) had values within the mid to high reference range (> 400 ng/L). Eleven iPLE dogs (45.8%) out of 24 had serum folate concentrations lower than the reference interval. Canine pancreatic lipase was measured in 21 iPLE dogs (55.2%) and it was increased in 4 of them (19%). All dogs with iPLE included in the study had gastrointestinal duodenoscopy performed. Twenty-six iPLE dogs (68.4%) had concurrent lower GI endoscopy in which the ileum was successfully intubated in 9 cases (23.7%). On histopathology, a predominantly lymphoplasmacytic infiltration of the intestinal mucosa was found in all iPLE dogs. With regard to the severity of lymphoplasmacytic infiltrate, mild (grade 1) duodenal, ileal and colonic histologic lesions were found in 0, 0, and 2 dogs, respectively; moderate (grade 2) duodenal, ileal and colonic histologic lesions were found in 18, 7, and 24 dogs, respectively; marked (grade 3) duodenal, ileal, and colonic histologic lesions were found in 20, 2, and 7 dogs, respectively. Dilated crypts with proteinaceous material and cellular debris (crypt abscesses) were identified in 9 iPLE dogs (23.7%). Lymphangiectasia was identified in 30 iPLE dogs (78.9%) and it was further classified as mild (*n* = 13, 43.3%), moderate (*n* = 15, 50%), and severe (*n* = 2, 6.7%). On endoscopy, pinpoint to coalescing white spots were found in 17 (56.6%) out of 30 iPLE dogs in which lymphangiectasia was detected histologically.

Prior to the admission 12 dogs with iPLE (31.6%) received highly digestible gastrointestinal diets, 2 dogs (5.2%) low fat diets, 12 dogs (31.6%) commercial limited ingredient diets, 4 dogs (10.6%) home-cooked selected protein diets, and 6 dogs (15.8%) hydrolyzed diets. Two dogs (5.2%) were fed different diet types. After T0, ultra-low fat and hydrolyzed diets were prescribed to 23 (60.5%) and 15 (39.5%) dogs, respectively. All iPLE dogs received oral prednisolone at the dose of 0.5 mg/kg twice daily. In addition, oral chlorambucil (2–4 mg/m2) was prescribed to 2 dogs (5.2%). Eighteen iPLE dogs received weekly parenteral cobalamin supplementation, 11 dogs received daily oral folate supplementation. Sixteen dogs received oral clopidogrel. Two dogs (5.2%) died for causes related to the iPLE between T0 and T1. Table 1 shows the selected clinicopathological variables of both populations at T0.

**TABLE 1:** Selected clinicopathological characteristics of healthy control dogs and dogs with iPLE at the time of the endoscopic procedure (T0).

Parameter	*N*	Median value	Min-max
**iPLE dogs**			
Age (months)	38	95.5	(19–171)
Body weight (Kg)	38	18	(3.5–42)
Sex (F/M) spayed/neutered	38 (19/19) 13/1	– –	–
CCECAI	38	8	(3–17)
Clinical disease severity groups (Allenspach et al., 2007)			
• Mild	5		
• Moderate	16		
• Severe	8		
• Very Severe	9		
TP (g/dL)	38	4.1	(2–7.4)
Albumin (g/dL)	38	1.8	(0.9–2.7)
Cholesterol (mg/dL)	36	116	(63–327)
CRP (mg/l)	34	4.6	(0–48.4)
Folate (ng/mL)	24	8.1	(2–24)
Cobalamin (pg/dL)	24	253	(62–1000)
**Healthy dogs**			
Age (months)	47	72	(18–204)
Weight (Kg)	47	18	(3.5–42)
Sex (F/M) spayed/neutered	47 (25/22) 22/13	– –	

*N*, Number of observations; CCECAI, canine chronic enteropathy clinical activity index; CRP, C-reactive protein; F, female; iPLE, inflammatory protein-losing-enteropathy; M, male; TP, total protein. Value, Data expressed as median (min-max).

At T1, the iPLE dogs showed the following median (range) values: CCECAI score 4 (0–13), total protein 5.1 g/dL (2.8–7.5), albumin 2.2 g/dL (1.3–3.1), cholesterol 138 mg/dL (66–237), and C-reactive protein 2.1 mg/L (0–31.4). Median CCECAI score was significantly decreased compared to that recorded at T0 (*p* = 0.001), while total protein and albumin were significantly increased (*p* < 0.0001). Cholesterol and C-reactive protein did not significantly change at T1.

Information regarding individual iPLE dogs is provided in Supplementary Table 1.

### 3.2 Dysbiosis Index (DI) and fecal metabolomics

**FIGURE 1:**
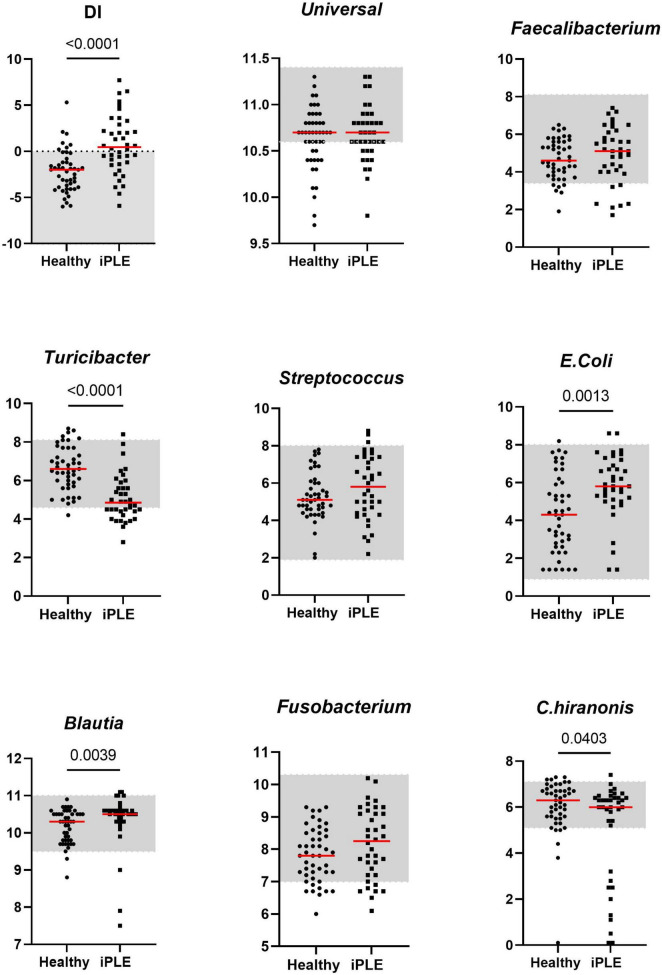
Comparison of Dysbiosis index (DI) and each taxa abundance between healthy control dogs and iPLE dogs at T0. Bacterial abundance is expressed as log DNA. The gray area represents the reference interval. Horizontal red lines represent medians. Only the significant *p*-values are shown in the graph.

The median DI was significantly higher in dogs with iPLE compared to healthy control dogs (*p* < 0.0001). The abundances of *E. coli* (*p* = 0.001) and *Blautia* (*p* = 0.004) were significantly higher in dogs with iPLE compared to healthy control dogs. The abundances of *C. hiranonis* (*p* = 0.041) and *Turicibacter* (*p* < 0.0001) were significantly lower in dogs with iPLE compared to healthy control dogs. All results are summarized in Figure 1 and Table 2.

**TABLE 2:** Summary statistic of the abundance of bacterial groups assessed for the calculation of the DI in healthy dogs and iPLE dogs.

	Healthy dogs median (range)	iPLE dogs median (range)	*p*-value	adjusted *p*-value
	*n* = 47	*n* = 38		
DI	−2.0 (−6.0–5.3)	0.4 (−5.9–7.7)	< 0.0001*	0.001*
Universal	10.7 (9.7–11.3)	10.6 (9.8–11.3)	0.468	4.21
*Faecalibacterium* spp.	4.6 (1.9–6.5)	5.1 (1.7–7.4)	0.388	3.49
*Turicibacter* spp.	6.6 (4.2–8.7)	4.8 (2.8–8.4)	< 0.0001*	0.001*
*Streptococcus* spp.	5.1 (2.0–7.8)	5.8 (2.2–8.8)	0.177	1.59
*E. coli*	4.3 (1.4–8.2)	5.8 (1.4–8.6)	0.001*	0.005*
*Blautia* spp.	10.3 (8.8–10.9)	10.5 (7.5–11.1)	0.004*	0.04*
*Fusobacterium* spp.	7.8 (6–9.3)	8.2 (6.1–10.2)	0.102	0.91
*C. hiranonis*	6.3 (0.1–7.3)	6.0 (0.1–7.4)	0.040*	0.36

Data are expressed as median (minimum−maximum) logDNA/gram of feces. *P*-value and adjusted *p*-value are set at 0.05 (95% confidence interval);

*statistically significant. DI, dysbiosis index; iPLE, inflammatory protein-losing enteropathy; *N*, numbers of observations.

Of the iPLE dogs, 11 (29%) had DI values > 2, indicating a severe shift in the microbiome. In 9 iPLE dogs (23.7%) the DI was mildly to moderately increased (0–2). Five iPLE dogs (13.1%) had normal DI values (< 2), but the abundance of individual bacteria was outside the respective reference ranges, indicating minor shift in the microbiome. DI values were normal without individual bacterial abundance changes in 13 iPLE dogs (34.2%). Of the healthy control dogs, 29 (62%) had normal DI values, while 18 (38%) had abnormal DI values with 7 dogs (15%) having a DI > 0 with 2 of these (4%) with a DI > 2 and one of them showed low *C. hiranonis* abundance; 11 healthy control dogs (23%) exhibited minor changes in the microbiome. No significant correlations were found between the DI and the following variables at T0: age, gender, body weight, CCECAI, serum concentrations of total protein, albumin, cholesterol, C-reactive protein, cobalamin, and folate.

**FIGURE 2:**
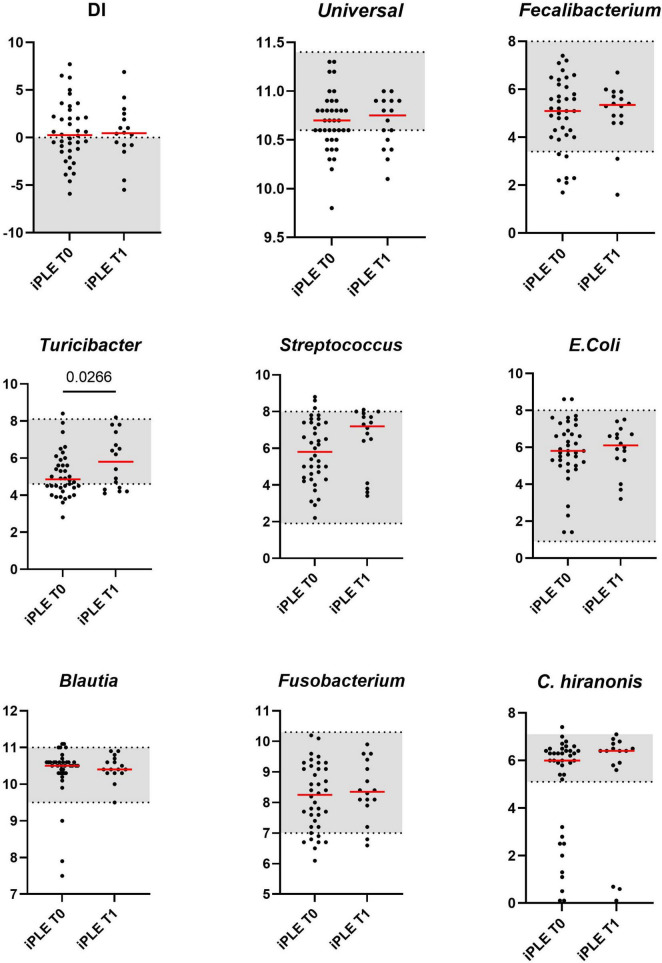
Comparison of Dysbiosis index (DI) and each taxa abundance of iPLE dogs between T0 and T1. Bacterial abundance is expressed as log DNA. The gray area represents the reference interval. Horizontal red lines represent medians. Only the significant *p*-values are shown in the graph.

Sixteen fecal samples were available for DI analysis at T1. The median DI value at T1 did not significantly differ from that at T0. The abundance of *Turicibacter* was significantly increased compared to that of T0 (*p* = 0.026). These results are shown in Figure 2. The classification of iPLE and healthy control dogs based on DI values at T0 and T1 is reported in Table 3. Nine out of 16 iPLE dogs exhibited a fold change indicating more than twofold decrease in the DI, with a fold change value less than 0.5. Only 1 iPLE dog showed a threefold increase in DI at T1 compared to T0.

**TABLE 3:** Classification of intestinal dysbiosis severity in healthy dogs and iPLE dogs based on DI values at diagnosis (T0) and after 1 month of therapy (T1).

	Healthy dogs (*N* = 47)	iPLE dogs at T0 (*N* = 38)	iPLE dogs at T1 (*N* = 16)
Significant dysbiosis	2	11	4
Mild to moderate changes	5	9	6
Minor changes	11	5	1
Normal	29	13	5

Significant Dysbiosis (DI > 2); mild to moderate changes (DI 0−2); minor changes (DI < 2, with individual bacterial groups outside the reference interval); normal (DI < 2, with no shifts in the overall diversity of the intestinal microbiota). iPLE, protein losing enteropathy; *n*, number of observations; T0 = diagnosis; T1, after 1 month of therapy.

#### 3.2.1 Fecal unconjugated bile acids concentrations

At T0, fecal concentrations of TPBA and their percentage (TPBA%) did not differ between healthy control and iPLE dogs. Similarly, TSBA and their percentage (TSBA%) did not significantly differ between healthy control and iPLE dogs. However, a subset of iPLE dogs (*n* = 12; 31.5%) showed increased fecal TPBA and decreased fecal TSBA, and 8 of them showed concurrent *C. hiranonis* reduction. Significant moderate negative correlations between the abundance of *C. hiranonis* and both TPBA and TPBA% (*r*_*s*_ = −0.48, *p* < 0.0001; *r*_*s*_ = −0.44, *p* < 0.0001) were found. Significant weak to moderate positive correlations between the abundance of *C. hiranonis* and both TSBA and TSBA% (*r*_*s*_ = 0.22, *p* = 0.043; *r*_*s*_ = 0.44, *p* < 0.0001) were found. Significant moderate negative correlations between DI and both TSBA and TSBA% (*r*_*s*_ = −0.54, *p* = 0.001; *r*_*s*_ = −0.50, *p* = 0.001) were found. Specifically, a moderate negative correlation was found between DI and lithocholic acid and deoxycholic acid (*r*_*s*_ = −0.55, *p* = 0.001; *r*_*s*_ = −0.53, *p* = 0.001). No significant correlation was found between each single unconjugated bile acid and the CCECAI score, serum albumin and serum cholesterol. The concentrations of both primary and secondary bile acids did not significantly differ among groups of dogs classified based on different types of diet fed prior to the admission.

No significant differences in fecal bile acids content were found at T1, compared to T0 in iPLE dogs. No significant differences were found in fecal primary and secondary bile acids content comparing dogs that were prescribed ultra-low fat diets and those that were prescribed hydrolyzed diets after T0.

**FIGURE 3:**
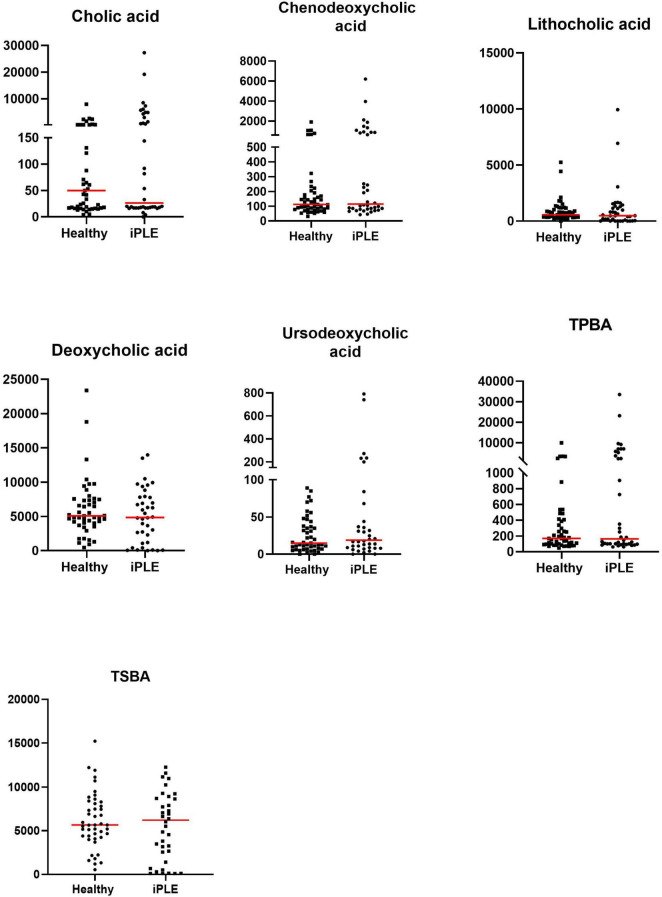
Fecal concentrations of unconjugated bile acids (ng/mg) in healthy control dogs and iPLE dogs. Red lines represent median value. Only the significant *p*-values are shown in the graph.

Summary statistics of fecal bile acid concentrations at T0 and T1 are shown in Figure 3 and Table 4.

**TABLE 4:** Summary statistics of fecal unconjugated bile acids, sterols and fatty acids concentrations in healthy control dogs and dogs with iPLE at diagnosis (T0) and after 1 month of therapy (T1).

Fecal unconjugated bile acids, sterols and fatty acids of healthy dogs and iPLE dogs at T0 (*n* = 38)				
	**Healthy dogs** **median (range)**	**iPLE dogs** **median (range)**	***p*-value**	**adjusted *p*-value**
**Unconjugated bile acids**				
Cholic acid (ng/mg)	50 (4–7985)	26.5 (0–27271)	0.416	1.000
Chenodeoxycholic acid (ng/mg)	113 (31–1910)	116 (43–6213)	0.230	1.000
Lithocolic acid (ng/mg)	558 (9–5252)	475 (5–9946)	0.274	1.000
Deoxycholic acid (ng/mg)	5104 (477–23381)	4871 (8–13988)	0.376	1.000
Ursodeoxycholic acid (ng/mg)	15 (0–89)	19 (0–791)	0.320	1.000
TPBA (ng/mg)	171 (48–9895)	166 (62–33485)	0.347	1.000
TSBA (ng/mg)	5649 (533–27901)	6201 (90–23979)	0.450	1.000
TPBA (%)	3 (1–95)	4.5 (1–98)	0.219	1.000
TSBA (%)	97 (5–99)	95.5 (2–99)	0.219	1.000
**Sterols**				
Coprostanol (μg/mg)	0.05 (0.01–0.34)	0.02 (0.01–20.10)	0.024*	0.840
Cholesterol (μg/mg)	2.28 (0.95–26.10)	7.34 (1.03–20.40)	<0.0001*	0.003*
Cholestanol (μg/mg)	0.30 (0.05–1.06)	0.18 (0.02–1.55)	0.060	1.000
Lathosterol (μg/mg)	0.01 (0.01–0.11)	0.06 (0.01–0.16)	<0.0001*	0.003*
Brassicasterol (μg/mg)	0.02 (0.01–0.07)	0.02 (0.01–0.23)	0.058	1.000
Campesterol (μg/mg)	0.31 (0.11–1.37)	0.25 (0.03–1.45)	0.032*	1.000
Stigmasterol (μg/mg)	0.17 (0.05–0.63)	0.14 (0.02–0.67)	0.021*	0.735
Fusosterol (μg/mg)	0.07 (0.02–0.28)	0.05 (0.01–0.30)	0.014*	0.490
Beta–sitosterol (μg/mg)	1.16 (0.33–4.08)	0.59 (0.02–4.30)	0.001*	0.035*
Sitostanol (μg/mg)	0.27 (0.03–1.49)	0.08 (0.01–1.26)	0.002*	0.070
Total measured sterols (μg/mg)	5.16 (2.49–27.80)	11.30 (3.04–26.40)	<0.0001*	0.003*
Total measured zoosterols (μg/mg)	2.71 (1.10–26.70)	9.35 (1.14–26.20)	<0.0001*	0.003*
Total measured phytosterols (μg/mg)	2.25 (0.64–7.78)	1.21 (0.13–7.82)	0.001*	0.035*
**Fatty acids**				
Myristic acid (μg/mg)	0.62 (0.26–2.60)	1.98 (0.41–8.12)	<0.0001*	0.003*
Palmitic acid (μg/mg)	4.81 (1.61–15.30)	16.90 (2.09–22.20)	<0.0001*	0.003*
Linoleic acid (μg/mg)	4.09 (1.42–20.10)	7.49 (2.25–28.90)	<0.0001*	0.003*
α-linolenic acid (μg/mg)	0.35 (0.08–5.23)	0.20 (0.05–2.65)	0.072	1.000
Oleic acid (μg/mg)	3.84 (1.35–20.20)	10.40 (3.41–22.20)	<0.0001*	0.003*
Cis-vaccenic acid (μg/mg)	0.93 (0.06–4.87)	7.07 (0.29–26.00)	<0.0001*	0.003*
Stearic acid (μg/mg)	2.19 (0.52–19.90)	8.35 (0.76–41.40)	<0.0001*	0.003*
Arachidonic acid (μg/mg)	1.65 (0.48–4.26)	3.75 (0.62–10.30)	<0.0001*	0.003*
Gondoic acid (μg/mg)	0.21 (0.09–0.76)	0.84 (0.19–5.52)	<0.0001*	0.003*
Docosonoic acid (μg/mg)	0.27 (0.12–1.26)	0.84 (0.21–5.08)	<0.0001*	0.003*
Erucic acid (μg/mg)	0.12 (0.04–0.66)	0.40 (0.10–8.64)	<0.0001*	0.003*
Nervonic acid (μg/mg)	0.32 (0.10–1.40)	1.21 (0.18–4.85)	<0.0001*	0.003*
Total measured fatty acids (μg/mg)	19.70 (6.99–64.8)	61.80 (12.90–136.00)	<0.0001*	0.003*
**Unconjugated bile acids**				
Cholic acid (ng/mg)	19 (4–19179)	128.5 (15–32721)	0.192	1.000
Chenodeoxycholic acid (ng/mg)	94 (43–3969)	256 (61–5794)	0.390	1.000
Lithocolic acid (ng/mg)	475 (6–1628)	774 (2–2905)	0.234	1.000
Deoxycholic acid (ng/mg)	4377 (14–9955)	5863 (6–11166)	0.211	1.000
Ursodeoxycholic acid (ng/mg)	19 (2–272)	31.5 (3–390)	0.291	1.000
TPBA (ng/mg)	106 (62–23148)	490 (78.00–38515)	0.524	1.000
TSBA (ng/mg)	5015 (101–11594)	7424 (55–13401)	0.211	1.000
TPBA (%)	3 (1–97)	8 (1.00–99)	0.329	1.000
TSBA (%)	97 (3–99)	92 (1–99)	0.329	1.000
**Sterols**				
Coprostanol (μg/mg)	0.02 (0.01–20.14)	0.02 (0.01–15.79)	0.106	1.000
Cholesterol (μg/mg)	6.21 (2.88–14.30)	4.38 (1.88–9.00)	0.055	1.000
Cholestanol (μg/mg)	0.21 (0.08–0.76)	0.22 (0.07–0.74)	0.695	1.000
Lathosterol (μg/mg)	0.06 (0.01–0.16)	0.03 (0.01–0.84)	0.064	1.000
Brassicasterol (μg/mg)	0.02 (0.01–0.07)	0.02 (0.01–0.06)	0.687	1.000
Campesterol (μg/mg)	0.23 (0.04–0.50)	0.28 (0.07–0.83)	0.252	1.000
Stigmasterol (μg/mg)	0.13 (0.02–0.40)	0.17 (0.05–0.74)	0.257	1.000
Fusosterol (μg/mg)	0.03 (0.01–0.28)	0.06 (0.01–0.24)	0.277	1.000
Beta-sitosterol (μg/mg)	0.44 (0.05–1.93)	1.17 (0.14–3.21)	0.223	1.000
Sitostanol (μg/mg)	0.07 (0.01–0.68)	0.12 (0.02–1.28)	0.182	1.000
Total measured sterols (μg/mg)	10.96 (5.38–26.44)	8.78 (4.20–20.10)	0.129	1.000
Total measured zoosterols (μg/mg)	9.75 (3.06–26.22)	6.17 (2.03–19.78)	0.104	1.000
Total measured phytosterols (μg/mg)	0.89 (0.21–3.32)	1.67 (0.3–5.94)	0.182	1.000
**Fatty acids**				
Myristic acid (μg/mg)	1.57 (0.41–5.62)	0.80 (0.45–4.81)	0.073	1.000
Palmitic acid (μg/mg)	15.08 (3.47–19.63)	11.47 (4.33–19.44)	0.231	1.000
Linoleic acid (μg/mg)	6.77 (3.01–10.16)	5.36 (2.81–34.02)	0.348	1.000
α-linolenic acid (μg/mg)	0.14 (0.07–1.92)	0.45 (0.09–2.12)	0.487	1.000
Oleic acid (μg/mg)	7.90 (3.47–18.37)	5.34 (2.76–19.44)	0.044*	1.000
Cis-Vaccenic acid (μg/mg)	6.72 (1.49–15.85)	2.63 (0.99–14.90)	0.104	1.000
Stearic acid (μg/mg)	8.16 (2.97–18.16)	5.70 (2.14–18.36)	0.013*	0.455
Arachidonic acid (μg/mg)	4.30 (1.37–10.25)	2.29 (0.95–5.29)	0.051	1.000
Gondoic acid (μg/mg)	0.67 (0.19–2.49)	0.42 (0.18–1.66)	0.140	1.000
Docosonoic acid (μg/mg)	0.93 (0.24–5.08)	0.69 (0.27–2.18)	0.226	1.000
Erucic acid (μg/mg)	0.39 (0.10–1.19)	0.24 (0.06–0.57)	0.018*	0.630
Nervonic acid (μg/mg)	1.18 (0.18–2.49)	0.67 (0.23–1.85)	0.002*	0.070
Total measured fatty acids (μg/mg)	53.36 (24.17–94.01)	39.86 (17.60–92.13)	0.083	1.000

H, healthy; iPLE, inflammatory protein-losing enteropathy; T0, diagnosis; T1, after 1 month of therapy; TPBA, total primary bile acids; TSBA, total secondary bile acids;

*statistically significant.

#### 3.2.2 Fecal long-chain fatty acids concentrations

**FIGURE 4:**
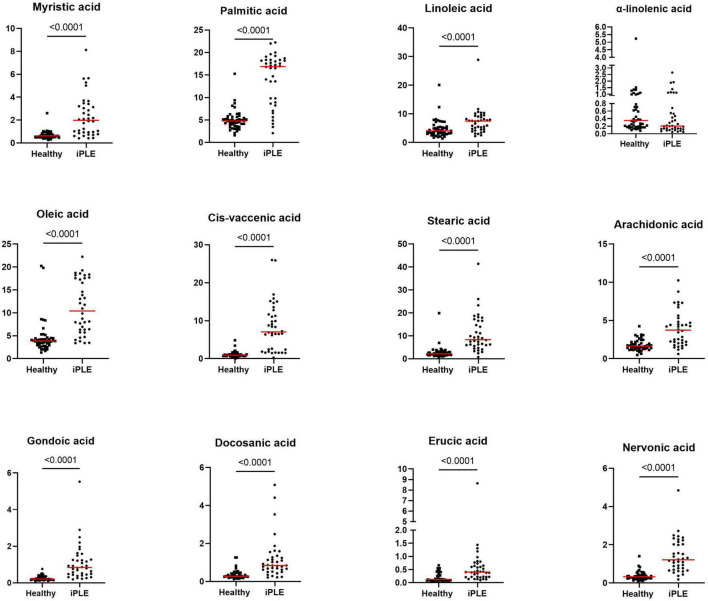
Fecal concentrations of long-chain fatty acids (μg/mg) in healthy control dogs and iPLE dogs. Red lines represent median value. The *p*-values expressed are unadjusted (adjusted *p*-value are reported in Table 4).

At T0, FAs and the concentration of each long-chain fatty acids measured, except α-linolenic acid, were significantly higher in dogs with iPLE compared to healthy control group (all *p* < 0.0001). The median α-linolenic acid concentration was lower in iPLE dogs compared to healthy control dogs, but this difference was not statistically significant. Summary statistics of FAs at T0 are shown in Figure 4 and Table 4.

FAs did not significantly differ between dogs with and without lymphangiectasia, nor between dogs with mild and moderate to severe lymphangiectasia. A significant weak to moderate negative correlation between total FAs and both serum total protein and albumin concentrations (*r*_*p*_ = −0.34, *p* = 0.036; *r*_*p*_ = −0.57, *p* = 0.001) was found; a significant moderate positive correlation with fecal cholesterol concentration (*r*_*s*_ = 0.55, *p* = 0.001) was found. Significant weak to moderate negative correlations between nervonic, cis-vaccenic, stearic, oleic, gondoic, myristic, palmitic acid and serum albumin (*r*_*p*_ = −0.37, *p* = 0.022; *r*_*p*_ = −0.57, *p* = 0.001; *r*_*p*_ = −0.46, *p* = 0.003; *r*_*p*_ = −0.53, *p* = 0.001; *r*_*p*_ = −0.48, *p* = 0.002; *r*_*p*_ = −0.58, *p* = 0.001; *r_*p*_* = −0.36, *p* = 0.024) were found.

**FIGURE 5:**
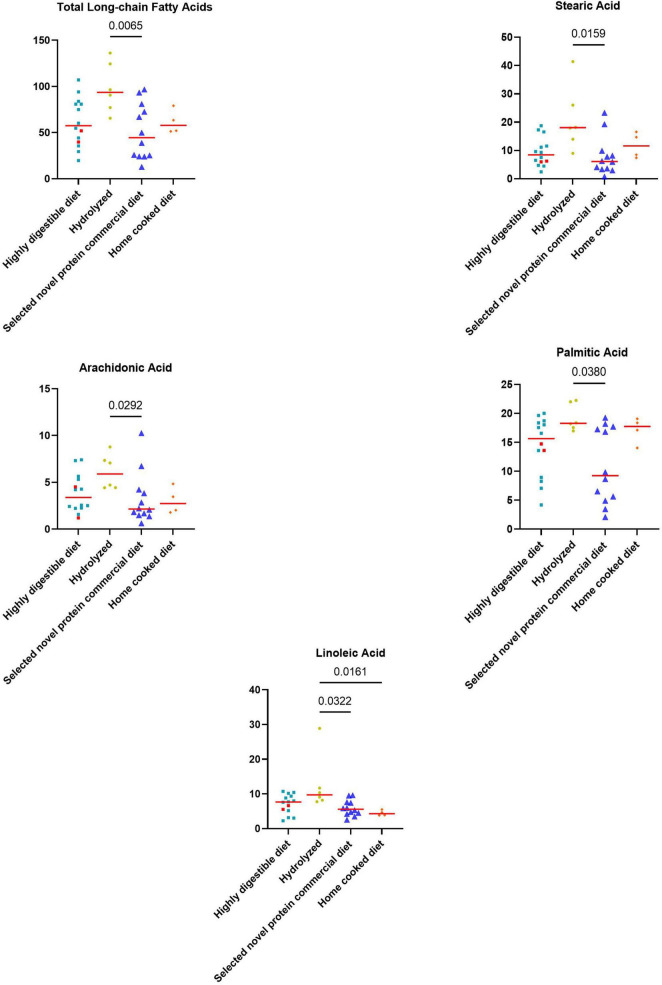
Fecal concentrations of metabolites that differed significantly between diet groups prior to the inclusion. The two iPLE dogs that were fed with highly digestible gastrointestinal low-fat diets are highlighted in red. Red lines represent median values. Significant *p*-values from the post-hoc analysis for multiple comparisons following the Kruskal-Wallis test are displayed.

Significant weak to moderate positive correlations between the CCECAI score and FAs (*r*_*s*_ = 0.32, *p* = 0.049), and concentrations of myristic acid (*r*_*s*_ = 0.34, *p* = 0.034), oleic acid (*r_*s*_* = 0.43, *p* = 0.006), cis-vaccenic acid (*r*_*s*_ = 0.35, *p* = 0.031) and gondoic acid (*r*_*s*_ = 0.42, *p* = 0.008) were found. No significant correlation was found between FAs and DI values, nor between DI and each long-chain fatty acid measured. Few correlations were found between long-chain fatty acids measured and single bacterial taxa. Specifically, a mild and positive correlation was found between nervonic, myristic, gondoic acid and *E. coli* (*r*_*s*_ = 0.35, *p* = 0.027; *r*_*s*_ = 0.38, *p* = 0.017; *r*_*s*_ = 0.34, *p* = 0.033), and a weak negative correlation was found between arachidonate and *Streptococcus* (*r*_*s*_ = −0.36, *p* = 0.026). FAs (*p* = 0.011), stearic acid (*p* = 0.022), palmitic acid (*p* = 0.040), linoleic acid (*p* = 0.010) and arachidonic acid (*p* = 0.039) were significantly higher in dogs that were fed hydrolyzed diets prior to the admission compared to dogs that received other diets (Figure 5).

**FIGURE 6:**
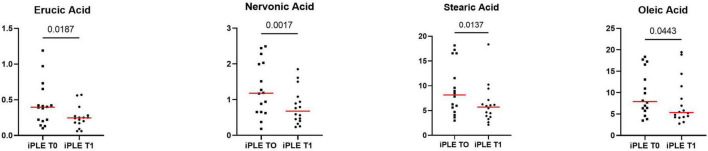
Fecal concentrations of selected metabolites that significantly improved at T1 in dogs with iPLE. Red lines represent median value. The *p*-values expressed are unadjusted (adjusted *p*-value are reported in Table 4).

At T1, the concentrations of oleic acid (*p* = 0.044), stearic acid (*p* = 0.013), erucic acid (*p* = 0.018) and nervonic acid (*p* = 0.002) significantly decreased (Figure 6 and Table 4). At fold change analysis some metabolites exhibited fold changes that were biologically meaningful. However, the analysis did not clearly identify a distinct subset of iPLE dogs that responds in a consistent manner to the treatment based on fecal metabolite changes. The results of the fold change analysis are presented in Supplementary Table 2. No significant differences in fatty acids (FAs) or individual long-chain fatty acids between dogs prescribed an ultra-low fat diet and those prescribed a hydrolyzed diet were found.

#### 3.2.3 Fecal sterols concentrations

**FIGURE 7:**
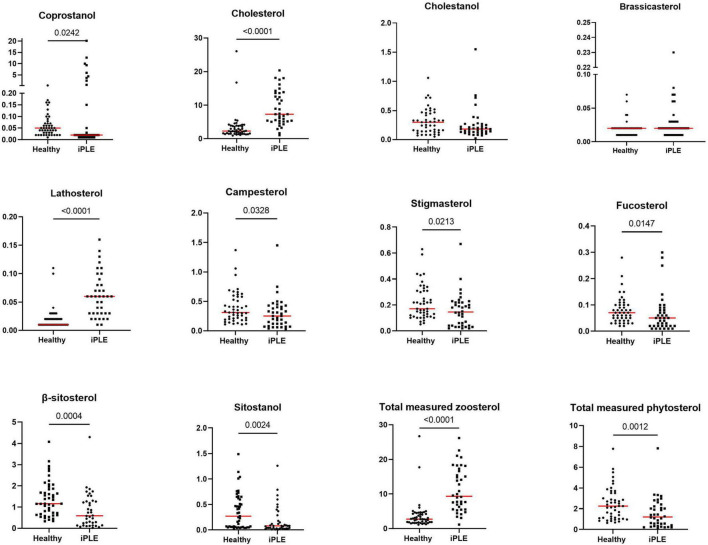
Fecal concentrations of sterols (μg/mg) in healthy control dogs and iPLE dogs. Red lines represent median value. The *p*-values expressed are unadjusted (adjusted *p*-value are reported in Table 4).

At T0, the concentration of total sterols was significantly higher in dogs with iPLE compared to that of healthy control dogs (*p* < 0.0001). The concentration of total zoosterols were significantly higher in dogs with iPLE compared to those of healthy control dogs (*p* < 0.0001), while total phytosterols were lower in iPLE than in healthy dogs (*p* = 0.001). Among the zoosterols, the concentration of cholesterol (*p* < 0.0001), and lathosterol (*p* < 0.0001) were significantly higher in dogs with iPLE, compared to healthy control dogs. In contrast, the concentration of coprostanol (*p* = 0.024) was significantly lower in iPLE dogs. The fecal concentration of cholestanol didn’t differ between iPLE dogs and healthy control dogs. The concentration of each measured phytosterol was significantly lower in dogs with iPLE compared to healthy control dogs, except for brassicasterol, which did not differ significantly. Summary statistics of sterols at T0 are shown in Figure 7 and Table 4.

No significant differences for the concentrations of both total phytosterols and zoosterols were found between dogs with and without lymphangiectasia, nor between dogs with mild and moderate to severe lymphangiectasia. No significant correlations were found between CCECAI sore and fecal concentrations of all measured sterols. No significant correlation was identified between DI values and the fecal concentrations of all measured sterols, except for a moderate negative correlation between coprostanol and DI (*r_*s*_* = −0.40, *p* = 0.012). However, when correlating fecal concentrations of coprostanol with individual bacterial taxa, no significant correlations were found. A weak and positive correlation was found between total phytosterols and *Streptococcus* (*r*_*p*_ = 0.32, *p* = 0.049). No significant correlations between each sterol measured and serum total protein, albumin, and cholesterol were found. The concentrations of phytosterols and zoosterols did not significantly differ among groups of dogs classified based on different types of diet fed prior to the admission.

At T1, neither total sterols, nor total phytosterols and total zoosterols differed significantly compared to T0. Individual sterols measured also did not differ between T0 and T1. At fold change analysis, some sterols exhibited fold changes that were biologically meaningful. However, the analysis did not clearly identify a distinct subset of iPLE dogs that responds in a consistent manner to the treatment. No significant difference was found in fecal phytosterols and zoosterols content comparing dogs that were prescribed ultra-low fat diets after T0 and dogs that were prescribed hydrolyzed diet.

#### 3.2.4 Fecal metabolites content

**FIGURE 8:**
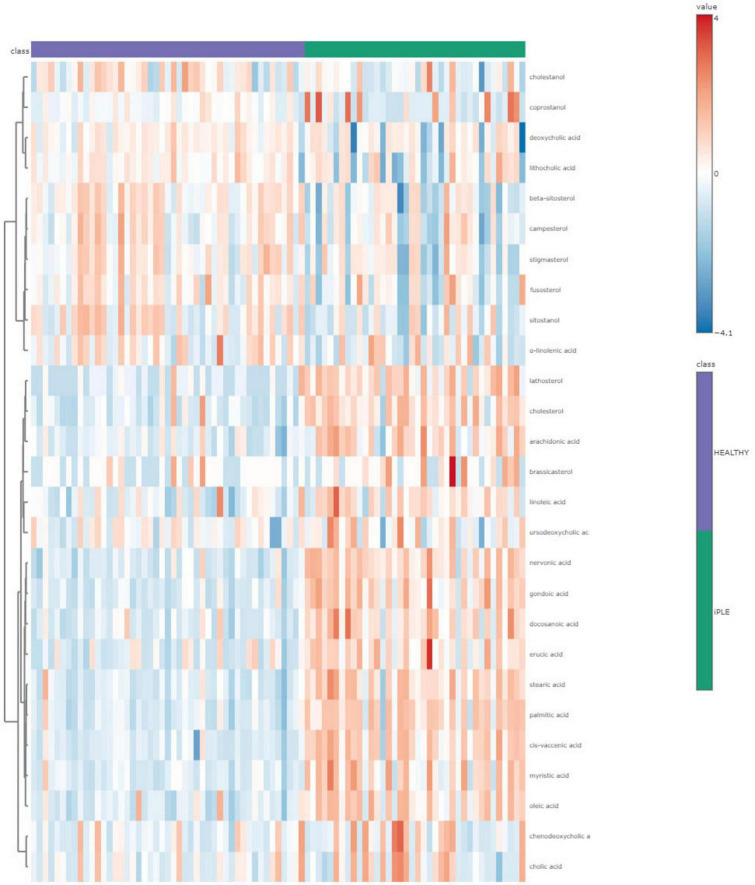
The heatmap provides intuitive visualization of the targeted fecal metabolites content differences among healthy control dogs and dogs with iPLE at T0. Each colored cell on the map corresponds to a concentration value, with samples in columns and metabolites in rows. The *t*-test was used by the software for the statistical analysis.

**FIGURE 9:**
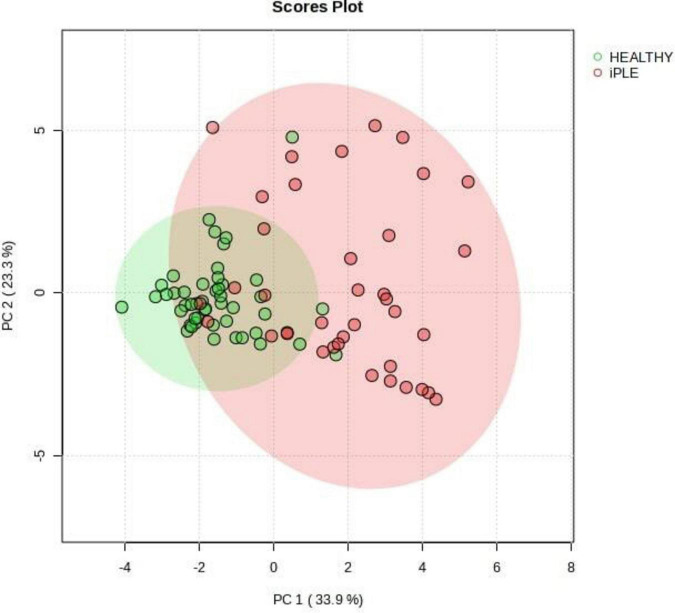
Principal Component Analysis (PCA) score plots of the targeted fecal metabolites for healthy control dogs and dogs with iPLE. The statistical significances of the group patterns are evaluated using PERMANOVA test (Permutational Multivariate Analysis of Variance). The figure shows healthy dogs cluster from iPLE dogs. PC1 = first principal component. PC2 = second principal component.

Figures 8, 9 show the comprehensive analysis of the differences in the targeted fecal metabolites content among healthy control dogs and dogs with iPLE.

## 4 Discussion

This prospective study aimed to evaluate intestinal dysbiosis and changes in fecal targeted metabolites in dogs with iPLE compared to healthy control dogs, and to investigate the impact of treatment on both the microbiome and the fecal metabolome in dogs with iPLE.

Several studies report a reduction of intestinal bacterial richness and diversity in a subset of dogs with gastrointestinal diseases, describing intestinal dysbiosis as a frequent finding in dogs with chronic enteropathy (Pilla and Suchodolski, 2020; AlShawaqfeh et al., 2017; Honneffer, 2017; Honneffer, 2014). Presence and severity of intestinal dysbiosis are not enough to differentiate the heterogeneous group of canine chronic enteropathies; however, the evaluation of both fecal microbiome and metabolome could reveal pathophysiologic mechanisms and potentially lead to new treatment strategies.

In this study, a qPCR-based index (DI) was employed to evaluate intestinal dysbiosis in dogs with iPLE. This quantitative assay is characterized by several advantages compared to sequencing techniques, including higher reproducibility, shorter analysis times, lower cost, and accessibility (AlShawaqfeh et al., 2017; Sung et al., 2023a; Sung et al., 2023b).

As expected, the median DI value was higher in dogs with iPLE compared to healthy dogs. However, 34.2% of dogs with iPLE had a normal DI value. This result might suggest that dysbiosis is a component of a more intricate pathogenetic process, but it is not a consistent finding in dogs with chronic intestinal inflammation, as also found elsewhere (AlShawaqfeh et al., 2017; Galler et al., 2022b). On the contrary, 29% of dogs with iPLE showed DI values > 2, that are indicative of severe dysbiosis and major shifts in the intestinal microbiome. This degree of dysbiosis is frequently observed both in case of severe gastrointestinal dysfunction and mucosal damage, and following antibiotic administration (Pilla and Suchodolski, 2020; Suchodolski et al., 2009; Pilla et al., 2020). Moreover, the effects of antibiotics on the microbiome last for several weeks and slowly decrease in the absence of chronic and persistent gastrointestinal disease in a subset of dogs (Stavroulaki et al., 2023). In this study, severe dysbiosis is presumed to be a consequence of the gastrointestinal mucosal damage and dysfunction, since iPLE dogs that received antibiotics in the 3 months prior to the admission were not enrolled. As expected, *C. hiranonis* abundance was significantly decreased in dogs with iPLE (AlShawaqfeh et al., 2017; Blake et al., 2019; Galler et al., 2022b) compared to the control group, and 26% of dogs with iPLE showed *C. hiranonis* abundance below the lower reference interval. *C. hiranonis* plays a key role in the conversion of primary to secondary bile acids and its decrease in fecal abundance leads to abnormal conversion of primary to secondary bile acid (AlShawaqfeh et al., 2017; Blake et al., 2019; Galler et al., 2022b; Manchester et al., 2019). In previous studies, it was shown that dysbiosis in dogs with chronic enteropathies, including some dogs with PLE, is often associated with shifts in the microbiome and reductions in some taxa such as *Faecalibacterium, Turicibacter*, and *C. hiranonis* (AlShawaqfeh et al., 2017; Sung et al., 2023a; Galler et al., 2022b). However, these changes are not consistent across all iPLE dogs, as there is a subset of iPLE dogs with clear shifts in the microbiome, and a subset of iPLE dogs that overlaps with healthy dogs both on 16S rRNA gene sequencing, DNA shotgun sequencing, and qPCR based assays (Sung et al., 2023a). This suggests some differences in the pathophysiology between these subsets that need to be further explored. Although the microbiome studies in dogs with PLE are still rare, it is evident that the similar subsets exist in dogs with PLE. In this study the DI was normal in a subset of dogs with iPLE, and in current (yet unpublished) dataset based on DNA shotgun sequencing the same results was evident that a subset of iPLE dogs clustered with the healthy controls. Therefore, future studies need to further explore the differences in underlying pathology between these subsets.

It is also interesting to note that some healthy control dogs, asymptomatic and not having a recent antibiotic history, were found to be dysbiotic (from mild to severe microbial shifts). These results suggest that microbial alterations are also present in dogs without gastrointestinal signs. However, only 2 healthy control dogs (4%) had a DI > 2. Nevertheless, subclinical intestinal inflammation of healthy control dogs was not ruled out with histopathologic examination of intestinal biopsies. In contrast to some previous studies (AlShawaqfeh et al., 2017; Galler et al., 2022a), a positive correlation between the CCECAI score and the DI value was not found here. This lack of correlation may be explained by the high CCECAI scores in all dogs with iPLE, with little inter-individual CCECAI variability.

Lastly, the results regarding serum cobalamin levels were available for 24 dogs with iPLE. The 50% of these dogs showed hypocobalaminemia and 25% showed suboptimal levels of cobalamin. All iPLE dogs with hypocobalaminemia and suboptimal levels of cobalamin were supplemented with parenteral or enteral cyanocobalamin. No significant correlation was found between the DI value and serum cobalamin concentrations. A recent study comparing the intestinal microbiome of dogs with and without hypocobalaminemia speculated that decreased serum cobalamin concentrations likely reflect the severity related to the underlying pathophysiology (Toresson et al., 2023). In light of this finding and considering that in this study only 29% of dogs with iPLE exhibited severe dysbiosis, the lack of correlation between hypocobalaminemia and dysbiosis might suggest that both low serum cobalamin concentrations and dysbiosis reflect the severity of mucosal damage and the disease, but they are not dependent variables.

Dogs with iPLE did not have any significant differences in fecal bile acid content compared to healthy control dogs. However, a subset of 31.5% of iPLE dogs had a concurrent increase in TPBA and a decrease in fecal TSBA content, suggesting reduced bile acid conversion in this subset of dogs with iPLE. These findings likely arose from bile acid malabsorption and intestinal dysbiosis. Bile acids are the main catabolic product of cholesterol metabolism with the important role of facilitating lipid digestion and absorption (Russell and Setchell, 1992). About 5–10% of primary bile acids are not reabsorbed in the ileum via the apical sodium-dependent bile acid transporter (Giaretta, 2018; Giaretta et al., 2018) and undergo conversion into secondary bile acids by the large intestinal microbiota. In dogs, *C. hiranonis* represents the main bile acid converter bacteria, having significant 7α-dehydroxylating activity (Blake et al., 2019; Manchester et al., 2019). Moreover, increased fecal primary bile acids could also be the consequence of decreased sodium-dependent bile acids transporter expression in the ileum and/or increased intestinal transit time (Giaretta et al., 2018).

In contrast to the beneficial effects of secondary fecal bile acids, primary bile acids are implicated in the dysregulation of the local immune and inflammatory response (Fiorucci et al., 2021; Gérard, 2013; Vítek, 2015). In this study *C. hiranonis* correlated negatively with primary bile acids and positively with secondary bile acids and its abundance was decreased in 8 out of 12 dogs that showed an increase in TPBA and a decrease in TSBA. These results support the role of *C. hiranonis* in the bile acid conversion and highlight the impact of intestinal dysbiosis in bile acid metabolism. A recent case report describe the successful use of bile acid sequestrants in dogs with chronic enteropathy that are non-responders to conventional therapeutic protocols (Toresson et al., 2021), suggesting bile acid malabsorption diarrhea as described in humans (Walters and Pattni, 2010; Camilleri, 2015). Therefore, the short-term use of bile acid sequestrants in some dogs with iPLE and bile acid dysmetabolism that are non-responsive to conventional therapy could be useful and deserves further investigation.

Marked perturbations in fecal long-chain fatty acid and sterol content of dogs with iPLE were found, indicating lipid malabsorption and dysmetabolism as previously described for dogs with chronic enteropathy and humans with inflammatory bowel disease (Piotrowska et al., 2021; Ma et al., 2019; Galler et al., 2022a; Honneffer, 2017). Increased fecal lipid content might be the result of different concurrent mechanisms, including altered epithelial transport as a result of damaged intestinal mucosa, reduced intestinal absorptive area and/or accelerated intestinal transit time. Long-chain fatty acids play an important role in maintaining the intestinal homeostasis, serving as antioxidants, and acting by various mechanisms that interfere with pro- and anti-inflammatory mediators (Wang et al., 2013). Moreover, their excesses in the gut lumen increase colonic motility and lead to osmotic diarrhea (Zhao et al., 2018). Arachidonic acid and its parent compound, linoleic acid, are omega-6 fatty acids that provide stability and fluidity to cell membranes. However, during periods of injury and inflammation, metabolism of arachidonic acid leads to the production of pro-inflammatory eicosanoids such as prostaglandin E2 thromboxane A2, and leukotriene B4 (Piotrowska et al., 2021). The increase in linolenic and arachidonic acid fecal content might suggest damage to the lipid bilayer of the intestinal epithelium and the presence of an active gastrointestinal inflammatory response in dogs with PLE. Palmitic acid affects intestinal epithelial and barrier integrity and permeability in in vitro studies (Gori et al., 2020), while nervonic acid could reflect depth and severity of the intestinal damage. Indeed, nervonic acid is an abundant component of the myelin sheath of nerves, and the muscularis and submucosal plexus are connected to the epithelium in the mucosa by enteric nerves and glial cells (Li et al., 2019). Moreover, nerves and glial cells that contain nervonic acid are also found in the mucosal layer (Ten Hove et al., 2021). A negative correlation between fecal content of nervonic acid and serum albumin concentration was found, further supporting the role of this fatty acid as a surrogate marker of intestinal damage. Nervonic acid is not commonly found within dietary components, making it unlikely that diet influences fecal nervonic acid content (Li et al., 2019). Even though the difference was not statistically significant, α-linolenic acid was the only long-chain fatty acid that was decreased in iPLE dogs. The α-linolenic acid is an omega-3 fatty acid with anti-inflammatory properties, acting as a competitive substrate for the enzymes and products of omega-6 polyunsaturated fatty-acid metabolism (Piotrowska et al., 2021). The omega-3/omega-6 imbalance possibly results in a disruption of the host’s immunity and overproduction of pro-inflammatory cytokines, perpetuating the inflammatory stimulus at intestinal level, similar to previous observations (Ma et al., 2019). A positive correlation between the CCECAI score and total FAs, along with some of the long-chain fatty acids measured was found, suggesting that the clinical severity may be associated with the severity of the intestinal damage. To the contrary, no significant correlation was found between FAs and DI values, but few weak correlations were found between *E. coli* and nervonic, gondoic and myristic acids, and between *Streptococcus* and arachidonic acid. These results suggest that dysbiosis does not influence the fecal content of long-chain fatty acids, and the few weak correlations found are insufficient to establish a clear role of dysbiosis in the observed metabolomic alterations. The relationship between *E. coli* and long-chain fatty acids is multifaceted and not well described (Mitchell and Ellermann, 2022); *E. coli* use long-chain fatty acids as an energy source and for membrane biosynthesis. At the same time, long-chain fatty acids can act as signaling molecules that influence *E. coli* virulence, particularly in pathogenic strains (Mitchell and Ellermann, 2022). However, further studies are needed to investigate the potential relationship between bacteria and metabolites.

At T1, a decrease in some long-chain fatty acids, including nervonic acid, was observed, although the values were still elevated compared to healthy dogs. This result might reflect the decrease in intestinal inflammatory stimulus and partial mucosal healing after treatment.

Sterols are a subclass of lipids embedded in cell membranes. They can be classified into two major groups: zoosterols, sterols of animal origin, and phytosterols, sterols of plant origin. In this study, dogs with iPLE showed a significant increase in fecal zoosterols and a significant decrease in phytosterols compared to healthy dogs. Similarly, recent analyses report decreased phytosterols in both dogs and cats with chronic enteropathy (Sung et al., 2023b; Honneffer, 2017). Phytosterols have anti-inflammatory properties, they can reduce cholesterol absorption in the gastrointestinal tract and they cannot be synthesized endogenously (Aldini et al., 2014; De Jong et al., 2003). Because of this, their fecal concentration is the end result of dietary ingestion and absorption. Therefore, malabsorption could be a reason for their increased fecal concentration. Moreover, unabsorbed phytosterols reach the colon and undergo bacterial metabolization; so it is reasonable to consider that dysbiosis may also play a role in phytosterol fecal content (Cuevas-Tena et al., 2018). However, in this study, we did not find a significant difference in phytosterol fecal content between dysbiotic and eubiotic dogs, and no correlation between fecal content of phytosterols and bacterial tax were found. Cholesterol is a key-role zoosterol, and it derives mainly from liver synthesis. Therefore, there is no absolute need for dietary intake, but regulation of the latter helps maintain a stable pool of cholesterol (Lecerf and de Lorgeril, 2011). In this study, fecal concentrations of cholesterol and lathosterol were significantly higher in iPLE dogs, contrary to coprostanol that was significantly decreased. Coprostanol is the non-absorbable end product of bacterial cholesterol metabolism (Kriaa et al., 2019). In this study, a significant negative and moderate correlation between the DI value and the coprostanol fecal content was found. Dogs with iPLE and normal DI values had higher fecal concentrations of coprostanol, compared to iPLE dogs with dysbiosis. This result could suggest reduced microbial conversion of cholesterol secondary to microbiota shift. Currently, human studies report *Eubacterium* and *Bacteroides* as main bacteria genera involved in cholesterol-to-coprostanol conversion, but other bacteria could exhibit similar properties (Gérard, 2013; Kriaa et al., 2019). The increase in fecal cholesterol concentration can result from various mechanisms acting simultaneously in dogs with iPLE. Firstly, it might increase due to the bile acid dysmetabolism. Secondly, enterocyte shedding from severe mucosal damage leads to an increased cholesterol concentration in the intestinal lumen, as it is a primary component of enterocyte membranes. Thirdly, cholesterol circulates esterified with fatty acids as lipoproteins in lymph vessels. In PLE disorders, intestinal inflammation leads to impaired drainage and altered permeability at the lacteal level, resulting in lacteal engorgement and rupture, with loss of proteins and lipids (cholesterol and fatty acids) into the intestinal lumen. Lastly, fecal cholesterol concentrations depend on animal diet sources, but we did not find any significant difference among different diet groups, nor at T0 or T1. In this study, no significant differences were found in the fecal concentrations of cholesterol between iPLE dogs with and without lymphangiectasia. These results, however, are not surprising since different factors can act simultaneously causing an increase in fecal cholesterol concentration, and because diet has a less important role on cholesterol compared to endogenous synthesis, as explained before. At T1, no significant changes were observed in fecal sterol content, in contrast to certain fatty acids, which decreased between T0 and T1. These fatty acids, however, did not return to levels comparable to healthy control dogs. This result possibly reflects a limited improvement in the aforementioned mechanisms with therapy, although one month of treatment may be too short to fully restore severe intestinal mucosal inflammation and damage.

In this study, the therapeutic and dietary protocols were not standardized, as the severity and chronicity of the disease, along with the alimentary history, dictated the choice of the therapeutic approach. For this reason, the changes in fecal lipid metabolism observed at T1 cannot be attributed to either dietary choices, use of anti-inflammatory/immunosuppressive medications, or a combination of both. Based on recent studies, dietary management with low fat or ultra-low fat diets seems to be the cornerstone of PLE treatment (Tolbert et al., 2022; Myers et al., 2023). Indeed, fat-restricted diets could break the cycle of intestinal protein and lipid leakage, decreasing lymphatic flow and lacteal distension. On the other hand, since some dogs with PLE have severe inflammation, pursuit of a hydrolyzed diet might be a valid therapeutic option (Green and Kathrani, 2022). In this study, fecal FA content (specifically, stearic acid, palmitic acid, linoleic acid, and arachidonic acid) was significantly higher in iPLE dogs that were fed a hydrolyzed diet prior to admission compared to dogs of other diet groups. Some hydrolyzed diets are soy-based, and soybeans have high content of stearic and linoleic acid.

Considering the overall results obtained in this study, it’s interesting to note that within the same population of dogs affected by the same gastrointestinal disease and with comparable clinical severity, there are subsets of iPLE dogs characterized by different microbiomic, metabolomic, and clinicopathological features. It’s possible to speculate that it’s not the disease itself that determines these alterations, but rather the severity of the intestinal mucosa damage. Dysbiosis, as well as hypocobalaminemia, are markers of underlying gastrointestinal damage, but they do not necessarily correlate with clinical disease activity. Conversely, certain fecal metabolomic perturbations (i.e., arachidonic acid, nervonic acid, and cholesterol) could serve as markers of disease activity and mucosal damage. However, this remains speculative at time, and further studies focused on this topic are needed.

This study is not without limitations. Firstly, due to ethical concerns, healthy control dogs did not undergo endoscopy to rule out the presence of asymptomatic gastrointestinal inflammation and lymphangiectasia. Secondly, follow-up fecal samples were not available for all iPLE dogs and only at 1 month after the histologic diagnosis. To ensure clinical relevance, it might be necessary to include more dogs at follow-up and consider a longer interval after the diagnosis. Last but not least, the dietetic and therapeutic approaches were not standardized, mainly due to different chronicity and severity of the disease among iPLE dogs, as explained above.

In summary, this study showed changes in the fecal microbiome and metabolome of dogs with iPLE. Notably, dysbiosis, bile acid, fatty acid, and sterol dysmetabolism were observed. Different therapeutic protocols lead to an improvement of some metabolome perturbations at short-term follow-up. Considering the important role of these compounds in inflammation and gastrointestinal tract functionalities, a comprehensive profile with long-chain fatty acids, sterols, and bile acids might help to better understand intestinal dysfunction in dogs with iPLE and possibly guide new therapeutic options in the future.

## Data Availability

The original contributions presented in the study are included in the article/Supplementary material, further inquiries can be directed to the corresponding author.
