# Supplementary material for: Dysbiosis index and fecal concentrations of sterols, long-chain fatty acids and unconjugated bile acids in dogs with inflammatory protein-losing enteropathy
Source: Front Microbiol. 2024 Oct 11;15:1433175. doi: 10.3389/fmicb.2024.1433175 (PMC11505111; doi:10.3389/fmicb.2024.1433175)
Supplement: Supplementary file 1 [file Table_1.docx]

Supplementary Table 1. Individual data of dogs with iPLE

|  | Age (months) | Sex | Breed | BW (Kg) | CCECAI  (T0) | Diet  (before T0) | Diet  (at T1) |
| --- | --- | --- | --- | --- | --- | --- | --- |
| #1 | 60 | FS | German Sheperd | 27 | 10 | Commercial limited ingredient diet | Hydrolyzed |
| #2 | 77 | M | Cavalier King Charles | 10 | 8 | Highly digestible GI diet | Ultra-low fat |
| #3 | 96 | M | Dachshund | 6.1 | 8 | Highly digestible GI diet | Hydrolyzed |
| #4 | 156 | M | German Sheperd | 47.5 | 4 | Commercial limited ingredient diet | Ultra-low fat |
| #5 | 19 | M | German Sheperd | 27 | 7 | Commercial limited ingredient diet | Hydrolyzed |
| #6 | 76 | M | Belgian Sheperd | 20.4 | 17 | Highly digestible GI diet | Ultra-low fat |
| #7 | 102 | FS | Mixed Breed | 10 | 3 | Highly digestible GI diet | Ultra-low fat |
| #8 | 127 | F | Mixed Breed | 14.5 | 10 | Highly digestible GI diet | Hydrolyzed |
| #9 | 156 | FS | Spanish Levriero | 15 | 7 | Commercial limited ingredient diet | Ultra-low fat |
| #10 | 60 | M | English Setter | 16.4 | 12 | Hydrolyzed | Hydrolyzed |
| #11 | 84 | M | Mixed Breed | 31.4 | 8 | Home cooked selected protein | Ultra-low fat |
| #12 | 132 | MC | Mixed Breed | 17.6 | 10 | Commercial limited ingredient diet | Ultra-low fat |
| #13 | 95 | M | Rottweiler | 37 | 9 | Different diet types | Ultra-low fat |
| #14 | 48 | M | Mixed Breed | 13.4 | 7 | Hydrolyzed | Hydrolyzed |
| #15 | 72 | M | Golden Retriever | 29 | 9 | Highly digestible GI diet | Ultra-low fat |
| #16 | 96 | F | German Sheperd | 22 | 12 | Home cooked selected protein | Hydrolyzed |
| #17 | 84 | FS | Border Collie | 13.7 | 14 | Hydrolyzed | Hydrolyzed |
| #18 | 90 | M | Mixed Breed | 10.9 | 8 | Commercial limited ingredient diet | Ultra-low fat |
| #19 | 132 | M | German Sheperd | 17 | 12 | Commercial limited ingredient diet | Hydrolyzed |
| #20 | 120 | FS | Cesky Terrier | 8.5 | 10 | Highly digestible GI diet | Ultra-low fat |
| #21 | 171 | FS | Maltese | 3.4 | 3 | Hydrolyzed | Hydrolyzed |
| #22 | 96 | F | American Staffordshire Terrier | 20.3 | 8 | Different diet types | Ultra-low fat |
| #23 | 6 | F | Mixed Breed | 9.8 | 8 | Commercial limited ingredient diet | Hydrolyzed |
| #24 | 108 | F | Belgian Sheperd | 23,7 | 7 | Highly digestible GI diet | Ultra-low fat |
| #25 | 110 | FS | Mixed Breed | 24.7 | 14 | Highly digestible GI diet | Ultra-low fat |
| #26 | 52 | F | Australian Sheperd | 22 | 8 | Hydrolyzed | Hydrolyzed |
| #27 | 24 | FS | German Sheperd | 20 | 12 | Commercial limited ingredient diet | Hydrolyzed |
| #28 | 96 | FS | Pittbull | 24.5 | 5 | Home cooked selected protein | Ultra-low fat |
| #29 | 70 | M | Yorkshire Terrier | 2.4 | 5 | Low fat | Ultra-low-fat |
| #30 | 84 | FS | French Bouledogue | 10 | 8 | Highly digestible GI diet | Hydrolyzed |
| #31 | 24 | M | Chihuahua | 2.65 | 12 | Highly digestible GI diet | Ultra-low fat |
| #32 | 60 | M | Rottweiler | 37 | 12 | Hydrolyzed | Ultra-low fat |
| #33 | 144 | M | Chihuahua | 3.9 | 8 | Commercial limited ingredient diet | Ultra-low fat |
| #34 | 120 | FS | Border Collie | 14.1 | 9 | Highly digestible GI diet | Ultra-low fat |
| #35 | 96 | FS | Podenco | 11.6 | 8 | Low fat | Ultra-low fat |
| #36 | 85 | M | Golden Retriever | 27.5 | 11 | Home cooked selected protein | Ultra-low fat |
| #37 | 108 | FS | Labrador Retriever | 35.4 | 7 | Commercial limited ingredient diet | Hydrolyzed |
| #38 | 119 | M | Border Collie | 25 | 8 | Commercial limited ingredient diet | Ultra-low fat |

BW=body weight; CCECAI=canine chronic enteropathy clinical activity index; F=Female; FS=female spayed; GI= gastrointestinal; iPLE=inflammatory protein-losing enteropathy; M=male; T0=diagnosis; T1=after 1 month of therapy
